# The association between sense of coherence and quality of life: a cross-sectional study in a sample of patients on hemodialysis

**DOI:** 10.1186/s40359-022-00805-9

**Published:** 2022-04-15

**Authors:** Toni Sawma, Yara Sanjab

**Affiliations:** 1 Department of Psychology, College of Sciences and Humanities, Effat University, Jeddah, Saudi Arabia; 2 Department of Psychology, School of Arts and Sciences, Holy Spirit University of Kaslik, Jounieh, Lebanon; 3 Department of Psychology, School of Arts and Sciences, Lebanese American University, Beirut, Lebanon; 4 Notre Dame Des Secours University Hospital, Psychology Unit, Street 93, Postal Code 3 Byblos, Mount-Lebanon, Lebanon

**Keywords:** Sense of coherence, Quality of life, Hemodialysis, Behavioral medicine

## Abstract

**Background:**

Patients on hemodialysis universally experience detriments in their general quality of life (QOL). Sense of coherence (SOC) is a long-standing factor that affects QOL. Nevertheless, the association between SOC and QOL in patients on hemodialysis was seldom investigated. Our research aims to study this association in a sample of Lebanese adult patients on hemodialysis.

**Methods:**

In this cross-sectional study, SOC was measured using the short version of the “Orientation of Life" scale (SOC-13). QOL was assessed using the Kidney Disease Quality of Life- Short Form (KDQOL-SF). The association between SOC and QOL and its submodalities was first explored using Pearson Correlation and multivariate linear regression analyses, controlling for sociodemographic variables and medical variables.

**Results:**

157 patients on hemodialysis (mean(SD) age: 62.1(13.81) years; 58.6% males) were included. Mean QOL score was 51.65 and that of SOC was 48.06. SOC was strongly corrected with the total QOL (r = 0.832; *p* value < 0.001), as well as its physical functioning (r = 0.767; *p* value < 0.001), emotional functioning (r = 0.757; *p* value < 0.001), cognitive functioning (r = 0.740; *p* value < 0.001), and social functioning (r = 0.773; *p* value < 0.001) submodailties. SOC was moderately correlated with professional functioning submodality of QOL (r = 0.618; *p* value < 0.001), but not correlated with the satisfaction with the quality of care (r = 0.052; *p* = 0.520). Within the regression models accounting for sociodemographic and medical variables, SOC was significantly associated with the total QOL (unstandardized B = 0.803; 95% CI 0.714, 0.893), physical functioning (unstandardized B = 0.761; standardized B = 0.763; 95% CI 0.661, 0.861), emotional functioning (unstandardized B = 1.205; standardized B = 0.757; 95% CI 1.037, 1.372), professional functioning (unstandardized B = 1.142; standardized B = 0.498; 95% CI 0.843, 1.442), cognitive functioning (unstandardized B = 1.239; standardized B = 0.739; 95% CI 1.058, 1.420), and social functioning (unstandardized B = 0.912; standardized B = 0.768; 95% CI 0.790, 1.034).

**Conclusions:**

In a sample of adult patients on hemodialysis, SOC was positively significantly associated with QOL and its submodalities, expect the satisfaction with the quality of care. The confirmation of the general hypothesis that relates the SOC with the QOL encourages more salutogenic research among this patient population.

## Introduction

End-stage kidney disease is a prevalent condition, with more than 2 million people receiving treatment worldwide [1]. Patients on hemodialysis experience substantial financial, professional, physical, and psychosocial hardships [2, 3], resulting in detriments in their general quality of life (QOL). The latter refers to one’s perceptions regarding their goals, expectations, standards, and concerns, as well as the position they hold in life within the cultural and value systems’ context in which they live. QOL is complexly influenced by one’s physical and psychological states, independence level, social relationships, and relationship with the salient characteristics of their environment [4]. It is generally believed that the QOL of patients on hemodialysis is poorer than that of age-matched subjects from the general population [5] and to that of patients receiving other forms of renal replacement therapy, especially renal transplantation [6].

QOL is increasingly being recognized as an outcome measure in the evaluation of dialysis therapy [7–9], whereby the aim is not only to improve survival, rather enhance the adjustment of patients to physical limitations, lifestyle changes, and medical interventions [10]. Improved QOL in patients on hemodialysis relates to positive outcomes, including decreased hospitalization and mortality [9, 11, 12]. Hence, the search for factors associated with improved QOL, i.e. salutogenesis, is gaining ground in the scientific literature.

Sense of coherence (SOC) is a long-standing factor that affects QOL. It is a global orientation to perceive the world and the individual environment as understandable, manageable, and meaningful [13]. SOC is a salutogenic concept created by Antonovsky to explain the cause of interindividual differences concerning successful adaptation following stress [14]. Comprehensibility is a person’s ability to create a structured representation of a situation that can be chaotic and to know the role a person has in resolving that situation [15]. The second behavioral component, i.e. manageability, comes to give the individual the ability to feel capable of change and to perceive the availability of the necessary resources, general or specific, to escape or face a specific situation [15]. The last component is the motivational component, i.e., meaningfulness. It gives motivation and a reason for overcoming issues that are seen as challenges, and helps to find the desire and the urge to invest energy to overcome a stressful experience [16]. Conceptually, chronic patients with a better comprehension are actively engaged in a cooperative relationship with the healthcare team. Moreover, patients with a better management can better identify and manage resources with optimism and perceive social support. Finally, patients who can find meaning to their disease are more motivated to pursue treatment [17]. Cross-sectional and longitudinal epidemiological data in patients with various diseases, like those with heart disease, cancer, and multiple sclerosis, as well as patients with kidney disease, such as those treated with home dialysis [18, 19], indicate that a stronger SOC relates to lower disease burden [20], survival quality [21–24], and a better perceived QOL [13].

Nevertheless, the independent association between SOC and QOL in patients on hemodialysis was seldom investigated; previous efforts mainly focused on patients in pre-dialysis [25], or those receiving peritoneal dialysis [26], in general through correlational studies. Exploring this association is expected to generate knowledge with implications for clinical practice, particularly therapeutic interventions to enhance SOC, ultimately leading to improve QOL.

The present study aims to assess the relationship between the SOC and the QOL in a sample of patients on hemodialysis in Lebanon. We hypothesize that a high SOC is associated with a better QOL.

## Method

### Study design

This was a cross-sectional study, conducted between November 2018 and February 2019, in three hospital-based hemodialysis units in the Mount Lebanon (n = 2) and North (n = 1) Governorates in Lebanon.

### Participants

The sample consisted of Lebanese, adult patients, receiving thrice-weekly hemodialysis treatment in three hospital-based units. All eligible patients receiving hemodialysis treatment in these units were approached to participate in the study. Patients receiving dialysis for acute kidney injury without chronic kidney disease, or those who are unconscious, suffering from dementia, under strong medication, i.e. high doses of psychotropic medications inducing sedative effects, or any condition that disable them from responding consciously to the questions, and those who have hearing problems were excluded.

### Sample size

Sample size calculation was conducted a priori on GPower 3.1. Using a medium effect size of 0.3, a two-tailed significance level at 0.01, and a power of 80%, the required sample size was 122. The chosen effect size was based on the reported correlation coefficient (r) between SOC and the mental component of QOL (r = 0.46) [27] in patients with chronic illness (end-stage renal disease, chronic heart failure, multiple sclerosis, stroke, and Parkinson), and that between SOC to QOL (r = 0.303) [28]. As per Cohen [29], an interval of (r) between 0.3 and 0.5 denotes a medium effect size. The initial sample size was increased by 42% to account for non-response, based on the response rate reported in a similar study (58%) [30], leading to a total of 174 patients.

### Outcome measures and assessment tools

Collected information included self-reported sociodemographic data (age, sex, level of education: uneducated, primary and elementary education, secondary education, and higher education), employment status: unemployed/retired, and employed, and marital status: single, married, and divorced/widowed), in addition to dialysis-related information (cause of kidney failure, duration on hemodialysis: 0–11 months, ≥ 12 months, the latter as a suggested time required for the patient to overcome the trauma of dialysis initiation and to psychologically adjust to dialysis [31], and previous transplants, when applicable), and medical data (use of psychiatric and non-psychiatric medications, number of hospitalization days in the last 6 months, and number of outpatient visits in the last 6 months), which were retrieved from the patients’ medical charts.

SOC was measured using the “Orientation of Life" scale developed by Antonovsky [16]. The short version of the scale containing 13 items (SOC-13) was used since it is recommended in settings such as hospitals and healthcare centers where it is difficult to use longer versions [32]. The SOC-13 has a well-established face validity, construct validity, consensual validity, and criterion validity in assessing SOC construct [13, 17], with good internal consistency (Cronbach's alpha coefficient = 0.70 to 0.92) [13]. SOC-13 includes 5 items for comprehensibility, 4 for manageability, and 4 for meaningfulness. The SOC-13 is rated using a 7-point Likert scale ranging between 1 and 7. The total score of the scale ranges between 13 and 91, with higher scores indicating a higher SOC. A SOC-13 score of 13–63 points corresponds to a low SOC, 64–79 points to a moderate SOC, and 80–91 points to a high SOC [33]. It takes about 6 min to be completed. The tool was translated into Arabic following recommended methods [34]. In details, the questionnaire was translated from English into Arabic by a translator. This was followed by a back-translation to English by another translator. This version was compared with the original English version to identify discrepancies. Then, the two translators made amendments to the Arabic version to produce the final version of the questionnaire.

QOL was assessed using the Kidney Disease Quality of Life- Short Form (KDQOL-SF) [35]. This questionnaire measures symptoms, effect of kidney disease on daily life, disturbance of kidney disease, cognitive functioning, professional status, sexual functioning, quality of social interactions, sleep, social support, and perceived encouragement from the healthcare team and patient satisfaction. These factors are divided into six submodalities: physical functioning, emotional functioning, cognitive functioning, professional functioning, social functioning, and satisfaction with the quality of care. The questionnaire includes 36 items measuring physical and mental health (short form of quality of life questionnaire), 43 kidney disease-targeted items (including two items for patients on peritoneal dialysis which were not considered in the current study), and one overall health rating item, leading to a total of 80 items. Each item was recoded to a 0–100 value, and relevant items were averaged to form each of the different submodalities The KDQOL-SF has well-documented reliability and construct validity for assessing QOL among dialysis patients, as well as a high internal consistency (Cronbach's alpha coefficient = 0.84 to 0.91) [36]. The Arabic validated version of the questionnaire [37] as used, which also shows statistically significant coefficients for reliability tests for all scales of the KDQOL-SF (0.492 to 0.936 for test–retest reliability and 0.337–0.994 for inter-observer reliability) and high internal consistency (Cronbach's alpha coefficient > 0.80). The KDQOL-SF takes about 16 min to be completed.

The Arabic versions of the questionnaires were pilot-tested on ten Lebanese patients on hemodialysis, the results of which were discarded. One psychologist helped the patients fill out the questionnaires during their stay in the dialysis center.

### Statistical analysis

Statistical analysis was conducted using the Statistical Package for Social Sciences (SPSS) version 21. Descriptive analyses were performed to summarize patients’ characteristics using means and standard deviation for continuous variables, and frequencies and percentages for categorical ones. The normality of distribution of the variables was confirmed by calculating their skewness and kurtosis; values for asymmetry and kurtosis between − 2 and + 2 were considered acceptable to prove normal univariate distribution [38]. The associations between sociodemographic variables and medical variables, with SOC and QOL were assessed using Independent-Samples T-Test for dichotomous categorical variables, and using Analysis of Variance (ANOVA) and Bonferroni post hoc or Kruskal–Wallis for categorical variables with at least three groups, as applicable. The association between SOC and QOL was first explored using Pearson Correlation, whereby a |r| between |0.90 and 1.00| denoted a very strong correlation, between |0.70 and 0.89| denoted a strong correlation, between |0.40 and 0.69| denoted a moderate correlation, between |0.10 and 0.39| denoted a weak correlation, and between |0.00 and 0.10| denoted a negligible correlation [39]. Then, seven multivariate linear regression analyses, using the Forward method, were used, entering QOL and each of its six submodalities, respectively as a dependent variable, and SOC as independent variable in addition to sociodemographic variables and medical variables with a p-value < 0.2 in the purposeful bivariate analysis [40–43], as well as variables of clinical or theoretical relevance, irrespective of their significance level in the bivariate analysis [44]. A two-tailed p-value < 0.05 was used as an indicator of statistical significance.

### Patient consent and ethical approval

Ethical approval was obtained from the Ethical Committee of the Notre Dame des Secours Centre Hospitalier Universitaire. Informed written consent was obtained from each patient before data collection. All the questionnaires were anonymous. The study was conducted according to the Declaration of Helsinki, and the psychology deontological rules were respected throughout the study.

## Results

During recruitment period, 196 patients were receiving treatment in the three hospital-based hemodialysis units; among whom, 25 patients were excluded as they were adolescents (n = 3), non-Lebanese (n = 2), receiving hemodialysis for acute kidney injury (n = 4), taking strong medications (n = 8), unconscious (n = 3), have dementia (n = 3), and have hearing problems (n = 2). Out of the 171 eligible patients, 157 consented to participate (response rate: 91.8%) and completed the questionnaires. As shown in Table 1, the average participants’ age was 62.1 years, more than half were males (58.6%), the majority were married (75%), unemployed or retired (72.3%), and receiving dialysis treatment for less than 1 year (84.1%). Finally, 5.7% of the sample had an unsuccessful kidney transplantation. and 26.8% were prescribed psychotropic medications.

**Table 1:** Characteristics of the study population

	N	%
Gender		
Male	92	58.6
Female	65	41.4
Marital status*		
Single	30	19.2
Married	117	75.0
Divorced/Widowed	9	5.8
Educational level		
Uneducated	13	8.3
Primary and elementary education	95	60.5
Secondary education	29	18.5
Higher education	20	12.7
Employment status		
Unemployed/Retired	114	72.6
Employed	43	27.4
Duration on hemodialysis		
0–11 months	132	84.1
≥ 12 months	25	15.9
Psychotropic medication		
No	115	73.2
Yes	42	26.8
Kidney transplantation		
No	148	94.3
Yes	9	5.7
Primary cause of kidney failure*		
Diabetes	30	19.4
Hypertension	21	13.5
Other	78	50.3
Does not know	26	16.8

	Mean	SD
Age	62.10	13.81
Inpatient days (last 6 months)	1.07	2.49
Outpatient visits (last 6 months)	0.20	0.59

^*^Valid percentages are presented due to missing values

SD: Standard deviation

The SOC and QOL of the study population are detailed in Table 2. The mean QOL score was 51.65 and mean SOC was 48.06.

**Table 2:** SOC, QOL, and its submodalities of the study population

	Total possible score	Mean	SD	Minimum	Maximum
SOC	91	48.06	15.91	20.00	87.00
QOL (total score)	100	51.65	15.92	14.93	85.75
Physical functioning	100	50.31	15.94	13.61	85.19
Emotional functioning	100	47.12	25.47	0.00	100
Professional functioning	100	40.45	36.75	0.00	100
Cognitive functioning	100	55.29	26.67	0.00	100
Social functioning	100	52.62	19.01		94.38
Satisfaction with the quality of care	100	89.15	18.15		100

SOC: Sense of coherence; QOL: Quality of life; SD: Standard deviation

As shown in Table 3, males, married patients, those with higher education, employed, having hypertension as primary cause of renal failure, and not prescribed psychotropic medications had higher QOL compared with their counterparts. Outpatient visits were weakly negatively correlated with QOL. The association between sociodemographic and medical variables with the submodalities of QOL are also detailed in Table 3.

**Table 3:** Association between sociodemographic and medical variables and SOC, with QOL and its submodalities

	QOL			Physical functioning			Emotional functioning			Professional functioning			Cognitive functioning			Social functioning			Satisfaction with the quality of car		
	Mean	SD	*p* value	Mean	SD	*p* value	Mean	SD	*p* value	Mean	SD	*p* value	Mean	SD	*p* value	Mean	SD	*p* value	Mean	SD	*p* value
Gender																					
Male	53.95	17.21	0.024	52.52	17.00	0.031	50.95	26.53	0.021	42.39	38.47	0.432	58.91	28.06	0.037	54.35	21.38	0.150	89.34	17.54	0.878
Female	48.39	13.36		47.17	13.83		41.69	23.00		37.69	34.27		50.15	23.84		50.17	14.85		88.89	19.13	
Marital status																					
Single	47.39	14.40	0.034	46.98	15.35	0.067	40.92	20.98	0.084	33.33	30.32	0.050	48.22	30.31	0.199	45.40*	18.60	0.009	86.02	22.75	0.850
Married	53.57*	16.16		52.01	16.08		49.74	26.22		44.02	37.81		57.55	25.93		55.33*	18.65		89.53	17.46	
Divorced/Widowed	42.70*	12.73		41.52	11.28		35.28	25.51		16.67	35.36		50.37	22.14		42.45	17.84		94.14	7.66	
Educational level																					
Uneducated	42.22†^	12.16	0.007	42.85	11.35	0.012 ±	32.50*‡	26.34	0.007	19.23*‡	25.32	0.006	36.41*‡	22.38	0.001	37.96*‡	15.45	0.006	89.32	18.39	0.715
Primary and elementary education	49.95	16.45		48.63	16.85		44.37	24.17		36.32	36.79		52.56⁋	25.74		51.75	19.57		90.23	17.23	
Secondary education	56.49†	12.50		54.33	12.60		55.69*	23.89		53.45*	32.54		62.30*	24.19		58.47*	13.78		88.31	19.01	
Higher education	58.86^	15.95		57.30	15.43		57.25‡	27.38		55.00‡	39.40		70.33‡⁋	28.07		57.79‡	20.26		85.14	21.62	
Employment status																					
Unemployed/Retired	48.33	15.00	< 0.001	47.10	14.98	< 0.001	42.92	25.08	0.001	31.58	32.08	< 0.001	50.82	26.23	0.001	49.97	18.91	0.004	89.04	17.70	0.894
Employed	60.46	15.08		58.80	15.44		58.26	23.26		63.95	38.31		67.13	24.36		59.64	17.62		89.47	19.51	
Duration on hemodialysis																					
0–11 months	52.25	16.07	0.278	50.79	16.15	0.387	47.61	25.59	0.577	43.18	36.95	0.032	56.82	26.62	0.098	53.32	18.54	0.291	89.60	17.32	0.477
≥ 12 months	48.47	15.06		47.77	14.82		44.50	25.14		26.00	32.66		47.20	25.96		48.92	21.36		86.78	22.28	
Psychotropic medication																					
No	53.38	16.69	0.011	52.03	16.79	0.010	49.46	25.90	0.057	43.91	36.96	0.050	57.97	27.12	0.029	53.89	19.56	0.165	88.50	19.17	0.458
Yes	46.91	12.62		45.59	12.33		40.71	23.36		30.95	34.84		47.94	24.21		49.13	17.15		90.94	15.09	
Kidney transplantation																					
No	51.21	16.01	0.161	49.87	16.04	0.169	46.64	25.57	0.340	40.20	37.19	0.738	55.23	26.78	0.907	51.93	18.84	0.067	88.81	18.52	0.342
Yes	58.89	13.26		57.42	12.98		55.00	23.68		44.44	30.05		56.30	26.27		63.87	19.36		94.75	9.05	
Primary cause of kidney failure																					
Diabetes	50.56	14.24	0.030	48.52	14.65	0.045	46.08	23.76	0.100	46.67	41.38	0.085	51.78	23.79	0.002	54.17	14.91	0.085	91.02	15.40	0.470
Hypertension	57.69*	16.66		56.63*	16.30		52.98	28.36		52.38	40.24		71.11*	23.79		56.28	16.15		83.33	25.05	
Other	52.84	16.46		51.39	16.83		49.46	25.76		39.74	34.53		56.75	27.22		53.82	19.57		90.28	17.59	
Does not know	44.46*	13.76		44.07*	12.84		36.63	22.10		26.92	32.34		42.05*	24.71		44.05	21.81		87.50	16.65	

	Correlation coefficient		*p* value	Correlation coefficient		*p* value	Correlation coefficient		*p* value		Correlation coefficient		*p* value		Correlation coefficient		*p* value	Correlation coefficient	*p* value	Correlation coefficient	*p* value
Age	− 0.127		0.114	− 0.164		0.04	− 0.075		0.348		− 0.053		0.510		− 0.052		0.515	0.040	0.616	0.014	0.866
Inpatient days (last 6 months)	− 0.046		0.571	− 0.030		0.717	-− .088		0.279		− 0.105		0.199		0.028		0.731	− 0.034	0.678	− 0.064	0.434
Outpatient visits (last 6 months)	− 0.245		0.002	− 0.224		0.005	− 0.247		0.002		− 0.281		< 0.001		− 0.108		0.177	− 0.207	0.009	0.010	0.901

^*^,^‡^,^⁋^Between-group significant difference using ANOVA and Bonferroni Post Hoc; ^†^,^Between-group adjusted significant difference using Kruskal–Wallis; ^±^No adjusted significant difference using Kruskal–Wallis

SOC: Sense of coherence; QOL: Quality of life; SD: Standard deviation

The results of the correlation between SOC, QOL, and each of its submodalities are detailed in Table 4. SOC was strongly corrected with the total QOL, as well as its physical functioning, emotional functioning, cognitive functioning, and social functioning submodalities. SOC was moderately correlated with professional functioning sub modality of QOL. Nevertheless, it was not correlated with the satisfaction with the quality of care.

**Table 4:** Correlation between SOC and QOL, and its submodalities

	SOC	
	Correlation coefficient	*p* value
QOL (total score)	0.829*	< 0.001
Physical functioning	0.757*	< 0.001
Emotional functioning	0.757*	< 0.001
Professional functioning	0.618*	< 0.001
Cognitive functioning	0.740*	< 0.001
Social functioning	0.767*	< 0.001
Satisfaction with the quality of care	0.052	0.520

^*^Correlation is significant at the 0.01 level

Table 5 presents the results of the linear regression analyses with QOL and each of its submodalities, expect satisfaction with the quality of care for which the multivariate analysis was not run, given the above-mentioned results. In all analyses, the association between SOC and the independent variable, i.e. QOL and each of its submodalities, remained statistically significant. Specifically, in the model considering QOL as dependent variable, the adjusted R Square of the model was 0.715, meaning that up to 70% of the variance of QOL is accounted for by the model. Accordingly, within this model accounting for potential sociodemographic and medical variables, SOC was highly significantly associated with QOL (unstandardized B = 0.803; standardized B = 0.806; *p* < 0.001; 95% CI 0.714, 0.893), whereby for a one-unit increase in SOC, a 0.8-unit increase in QOL is expected. Similarly, SOC was highly significantly associated with physical functioning, emotional functioning, professional functioning, cognitive functioning, and social functioning.

**Table 5:** Multiple linear regression analysis of SOC in relation to QOL

	Unstandardized B	Standardized B	95% CI		*p* value
			Lower	Upper	
Dependent variable: QOL					
SOC	0.803	0.806	0.714	0.893	< 0.001
Age	− 0.179	− 0.154	− 0.278	− 0.080	< 0.001
Employment status (Ref. unemployed/retired)	3.327	0.094	0.152	6.502	0.040
Adjusted R Square: 0.715 Variables included in the model: Age; Gender; Marital status; Educational level; Employment status; Duration on hemodialysis; Use of psychotropic medications; Kidney transplantation; Primary cause of kidney failure; Outpatient visits; SOC					
Dependent variable: Physical functioning					
SOC	0.761	0.763	0.661	0.861	< 0.001
Age	− 0.222	− 0.191	− 0.338	− 0.105	< 0.001
Adjusted R Square: 0.604 Variables included in the model: Age; Gender; Marital status; Employment status; Duration on hemodialysis; Use of psychotropic medications; Kidney transplantation; Primary cause of kidney failure; Outpatient visits; SOC					
Dependent variable: Emotional functioning					
SOC	1.205	0.757	1.037	1.372	< 0.001
Age	− 0.211	− 0.111	− 0.411	− 0.010	0.039
Adjusted R Square: 0.580 Variables included in the model: Age; Gender; Marital status; Educational level; Employment status; Duration on hemodialysis; Use of psychotropic medications; Kidney transplantation; Primary cause of kidney failure; Inpatient days; Outpatient visits; SOC					
Dependent variable: Professional functioning					
SOC	1.142	0.498	0.843	1.442	< 0.001
Employment status (Ref. unemployed/retired)	20.194	0.246	9.685	30.705	< 0.001
Duration on hemodialysis (Ref. 0–11 months)	− 15.068	− 0.150	− 27.462	− 2.675	0.018
Outpatient visits	− 8.705	− 0.138	− 16.523	− 0.887	0.029
Adjusted R Square: 0.452 Variables included in the model: Age; Marital status; Educational level; Employment status; Duration on hemodialysis; Use of psychotropic medications; Primary cause of kidney failure; Inpatient days; Outpatient visits; SOC					
Dependent variable: Cognitive functioning					
SOC	1.239	0.739	1.058	1.420	< 0.001
Adjusted R Square: 0.543 Variables included in the model: Age; Gender; Marital status; Educational level; Employment status; Duration on hemodialysis; Use of psychotropic medications; Primary cause of kidney failure; Outpatient visits; SOC					
Dependent variable: Social functioning					
SOC	0.912	0.768	0.790	1.034	< 0.001
Adjusted R Square: 0.587 Variables included in the model: Age; Gender; Marital status; Educational level; Employment status; Duration on hemodialysis; Use of psychotropic medications; Kidney transplantation; Primary cause of kidney failure; Outpatient visits; SOC					

## Discussion

Through multivariate analyses, this study showed a strong robust association between SOC and QOL and its submodalities, except the satisfaction with the quality of care, in a sample of patients on hemodialysis. Being on disease side of the health-disease continuum, patients receiving hemodialysis are likely to have a low to moderate SOC, given that SOC is initially a predictor of health [45]. This was also the case in our study, considering the low reported mean SOC.

Our finding of a positive association between SOC and physical functioning is also reported in individuals with chronic pain [46] and could be attributed to the role of SOC in modifying the long-term effects of diseases on the functioning of individuals [47]. It is plausible that people who have a high SOC can perceive their resources and consider themselves "good", even if they are in a state of illness [46]. Additionally, these individuals may, as they find meaning in their existence, also find meaning in the physical symptoms and pain they experience, leading to increased functioning.

The SOC is positively associated with emotional functioning. A similar finding was reported in uremic patients, not yet receiving dialysis [25], whereby the stronger the SOC, the less perceived anxiety. Termed as “emotional coherence”, the interplay between SOC and emotional functioning positively influences well-being [48, 49]. Individuals with a high SOC have more resources and the capacity to mobilize these resources to manage their emotions, while a weak SOC is significantly related to greater anxiety- especially somatic anxiety [32]. Also, a low SOC predicts depression [50] and the two concepts have shared characteristics [47, 51]. Yet, a high SOC does preclude depression. While SOC is likely to be stable throughout life, depression may ensue progressively, provided that factors conducive to depression outweigh the impact of SOC.

Our results regarding the positive association between SOC and professional functioning are in line with those of Schnyder et al. [47], where social and emotional dysfunction predicted deterioration of professional functioning. Individuals with a high SOC maintain acceptable functioning after trauma, adversity, or diagnosis compared with individuals with a low SOC [52]. This resides in their ability to take on professional tasks and view them as challenges, not stressors [53], hence finding pleasure in working with stressful demands and showing commitment and involvement in tasks without exhibiting symptoms of burnout [53]. Individuals who contribute at work have a greater capacity to mobilize their general resources of resistance to find a new meaning of their work and carry it on [17]. All of the above explain the recurrent finding in previous research and in our study that, among all the components of SOC, the meaning component is the most associated with professional functioning.

We also found that SOC is positively associated with cognitive functioning. Individuals who seek to adapt to changing conditions must be able to rely on their own ability to plan and solve problems [54]. This requires cognitive and executive functions, whereby an individual with a high SOC is expected to also have a relatively acceptable cognitive functioning.

Similar to the case of emotional functioning, SOC and social functioning are interplayed, whereby social support helps in the development of the SOC [49]. Drageset et al. [55], did not only find a positive correlation between the SOC and social functioning among residents of retirement homes, but also reported SOC as modifying factor in the relationship between social functioning and QOL. SOC was significantly and positively correlated with all the domains of QOL assessed by the Short-Form-36 questionnaire. Nevertheless, social support was positively correlated with health-related QOL irrespective of the level of SOC.

Amongst all submodalities of QOL, SOC was only not correlated with the satisfaction with the quality of care. The relationship between the dialysis patient and the healthcare team is unique, as it is continuous and includes issues of dependency, regardless of the internal resources and skills of the patient [56]. In addition, the patient does not perceive the relationship with the healthcare team as a purely therapeutic relationship; it is closer to the primary attachment relationship with the love object which is characterized by absolute dependence, but is now necessary to maintain life. Detachment from the caring body, outside the transplant, means death [57]. To make this debt life, the patient cannot disqualify the healthcare team, but on the contrary, idealizes it. There is an unconscious fear that caregivers would no longer provide care in case the patient gives them comments [58]. Hence, even patients with a weak SOC cannot criticize their healthcare team. Our results are similar to those reported by Langius and Lind [59], who highlight the lack of relationship between the SOC and the coordination or perception of information from the healthcare team among patients with oral and pharyngeal cancer.

The positive association between SOC and QOL have been long reported among chronic patients, such as those with Parkinson’s disease, chronic obstructive pulmonary disease, asthma, diabetes, cancer, and chronic heart diseases [51, 60–62], and in longitudinal studies, SOC was shown to predict better QOL [63]. Specifically, Delgado [64], found in patients on hemodialysis that a high SOC is associated with a better subjectively measured QOL and all its domains. However, such an association was not observed in acute dialysis patients, who typically do not develop a strong attachment with the medical team and do not feel the need to develop it since their dialysis therapy is of a short term [61].

Finally, besides its positive effect on QOL, SOC appears to protect against mortality. In a large population-based study (n = 20,579), a strong SOC was associated with a 30% reduction in mortality from all causes irrespective of gender, age, and chronic disease prevalence; this can be explained by the health resilience that a high SOC provides. This concept is primarily a tool for coping with stress; the lack of which predicts a high mortality rate [65].

### Clinical implications

Based on the findings of this study, it could be suggested that enhancing SOC would likely enhance QOL, opening for important therapeutic implications. especially the communication and interaction between the psychologist in the hospital, the medical team, the hospital, and the patient. It is highly plausible that enhancing SOC would result in a better QOL. Indeed, a recent pre-test–post-test control group experimental study among patients on peritoneal dialysis showed that salutogenesis-based care enhances the SOC and self-efficacy, provides symptom control, and decreases dialysis symptoms [26]. Specifically, through support therapy, psychologists are encouraged to listen, encourage, validate, and counsel. This will help the patient understand what he came through and accept that he has the right to have emotions and thoughts, i.e. enhance the comprehension component of the SOC. A psychologist can also target the management component of the SOC by helping the patient to identify their internal and external resources to become aware of their capacities to manage their situation and sustain their efforts, and to find a sense for their experience to realize that they are first and foremost persons, regardless of their diagnosis and illness [66], hereby improving patient’s meaning, i.e. the third component of the SOC. These components can also be targeted through other approaches, such as psychodynamic therapy [67] and cognitive behavioral strategies [68–70].

The healthcare team can also play a decisive role in the development of a SOC in patients in hospitals and nursing homes for a better QOL. The nursing team should show a constant and sufficient form of care. Furthermore, clear and coherent communication activates understanding among those receiving care. Caregivers can use communication tools to get the message across, such as the "teach-back", ultimately leading to a good understanding [71]. They can also help and encourage the patient to become aware of their resources and to use them routinely in a salutogenic way; hence elevating their sense of management. Likewise, the emotional and social support presented by the nursing body will, in addition to continual motivation, lead to a better meaning of life. A sense of belonging can also be developed, especially in departments where contact is continuous with the nursing staff, such as in the case of hemodialysis [55].

Hospitals are seen as formal resources for patients. Their architecture must consider this role. The availability of resources offered by the hospital architecturally is a factor that helps the patient psychologically [72]. The ergonomic architecture of hospitals that takes into account the needs of the patient can increase the speed of healing by up to 30.8% and decrease mortality by 38% [17]. Enhancing the "functionality" of the care space improves the SOC, specifically control, for the patient and the healthcare team simultaneously. With clear signals and informative papers, the hospital structure can enhance the comprehension of the patient [72]. Even if the hospital is not the ideal place to help the patient find meaning in life, the ergonomics of the environment can be inspiring and can create facilitations to decrease the isolation and the non-productivity of the patients during their hospitalization [72].

### Strengths and limitations

This study pioneers in exploring the association between SOC and QOL and its submodalities in the Arab World, specifically Lebanon. This article hence addresses a research priority and contributes to filling the research gap in mental health in the Arab World [73, 74]. This study was conducted on a powered sample, using validated and widely used tools in the international literature to assess the variables of interest, namely, the SOC-13 and KDQOL-SF. Furthermore, while the association between SOC and QOL was previously in general assessed using bivariate statistics, we explored this relationship using multivariate statistics accounting for numerous sociodemographic and clinical characteristics.

In contrast, we acknowledge the limitation that some factors associated with QOL among patients on hemodialysis were not included in the present study, such as coping strategies, resilience, religiosity…, and that we opted for self-reported data for a limited number of variables. Also, since a psychologist helped in collecting the data from the patients, this would have introduced some social acceptability bias. Furthermore, given the cross-sectional nature of this research, a cause-and-effect relationship between the SOC and QOL cannot be established. Whilst this study provides evidence on an association between these two variables, and robust interventional studies are needed to establish the hypothesis that enhancing SOC could indeed enhance the QOL of patients on hemodialysis. Moreover, further research is needed to ascertain if SOC is conceptually different from self-efficacy, hope, and resilience. This is an important question to address specifically in this patient-population, especially that, for example, studies have shown that hope is associated with a decreased burden of fluid and dietary restriction in dialysis patients [75] and is associated with a decreased incidence of the decline in physical function [76].

It is worth noting challenges encountered during the execution of this study: collecting information from the patients was challenging, especially since some included questions enhance the awareness of the patients about their daily difficulties, which may be accompanied by an emotional discharge. The processing time was also limited by the fatigue of the patients and their snack time, which must be respected in their case. All of these are well-known challenges when working with patients on hemodialysis, and anticipating them can enhance the data collection process.

Finally, while not recruited in a random manner, our sample resembles the general population of patients treated with hemodialysis in Lebanon [77] in many sociodemographic aspects, such as age and gender distribution, marital status, employment status, as well as clinical aspects such as the use of psychotropic medications and failed kidney transplant, potentially enabling us to generalize our findings to this population. Although the Lebanese society is characterized by its resilience, and familial [78], as well as its social support [79]- all of which are known actors associated with SOC and QOL in chronic patients, we suggest that our findings could be generalizable to other settings. because according to Antonovsky and Saggy [80], there is a “cultural stability” of SOC, and the higher the SOC, the more it mobilizes cultural stability. This concept of cultural stability of SOC is evident in the scientific literature [81, 82].

## Conclusion

In a sample of patients on hemodialysis, SOC was highly significantly associated with QOL and its submodalities, except the satisfaction with the quality of care. Longitudinal studies investigating means to enhance SOC, through supportive therapy, psychodynamic interventions, or cognitive/behavioral strategies, and exploring its effect on QOL and its submodalities are needed. Also, future studies should look into mediating factors between SOC and QOL in chronic patients, such as coping.

## Data Availability

All data analyzed during this study are included in this published article.

## Abbreviations

ANOVA: Analysis of Variance
KDQOL-SF: Kidney Disease Quality of Life- Short Form
QOL: Quality of Life
SD: Standard deviation
SOC: Sense of Coherence
SOC-13: Orientation of Life scale
SPSS: Statistical Package for Social Sciences

## References

[1] National Kidney Foundation. Global Facts: About Kidney Disease: National Kidney Foundation; 2021 [26–06–2021]. Available from: https://www.kidney.org/kidneydisease/global-facts-about-kidney-disease.

[2] Brown EA, Zhao J, McCullough K, Fuller DS, Figueiredo AE, Bieber B (2021). Burden of kidney disease, health-related quality of life, and employment among patients receiving peritoneal dialysis and in-center hemodialysis: Findings from the DOPPS program. Am J Kidney Dis.

[3] House TR, Wong SP (2021). What Is the “Maintenance” in Maintenance Dialysis?. Am J Kidney Dis.

[4] WHOQoL Group. The development of the World Health Organization quality of life assessment instrument (the WHOQOL). Quality of life assessment: International perspectives: Springer; 1994. p. 41–57.

[5] Cleary J, Drennan J (2005). Quality of life of patients on haemodialysis for end-stage renal disease. J Adv Nurs.

[6] Maglakelidze N, Pantsulaia T, Tchokhonelidze I, Managadze L, Chkhotua A (2011). Assessment of health-related quality of life in renal transplant recipients and dialysis patients. Transpl Proc.

[7] Chong K, Unruh M (2017). Why does quality of life remain an under-investigated issue in chronic kidney disease and why is it rarely set as an outcome measure in trials in this population?. Nephrology Dialysis Transpl.

[8] Nair D, Wilson FP (2019). Patient-reported outcome measures for adults with kidney disease: current measures, ongoing initiatives, and future opportunities for incorporation into patient-centered kidney care. Am J Kidney Dis.

[9] Valderrábano F, Jofre R, López-Gómez JM (2001). Quality of life in end-stage renal disease patients. Am J Kidney Dis.

[10] Zazzeroni L, Pasquinelli G, Nanni E, Cremonini V, Rubbi I (2017). Comparison of Quality of Life in patients undergoing hemodialysis and peritoneal dialysis: a systematic review and meta-analysis. Kidney Blood Press Res.

[11] Hall RK, Luciano A, Pieper C, Colón-Emeric CS (2018). Association of Kidney Disease Quality of Life (KDQOL-36) with mortality and hospitalization in older adults receiving hemodialysis. BMC Nephrol.

[12] Kalantar-Zadeh K, Kopple JD, Block G, Humphreys MH (2001). Association among SF36 Quality of Life measures and nutrition, hospitalization, and mortality in hemodialysis. J Am Soc Nephrol.

[13] Eriksson M, Lindström B (2007). Antonovsky's sense of coherence scale and its relation with quality of life: a systematic review. J Epidemiol Community Health.

[14] Breault J. Traduction de l'échelle du sens de cohérence et vérification de ses qualités psychométriques: École des hautes études commerciales; 2005.

[15] Volanen SM. Sense of coherence: Determinants and consequences. Helsingin Yliopisto; 2011.

[16] Antonovsky A. Unraveling the mystery of health: How people manage stress and stay well: Jossey-bass; 1987.

[17] Mittelmark MB, Sagy S, Eriksson M, Bauer GF, Pelikan JM, Lindström B (2017). The handbook of salutogenesis.

[18] Ageborg M, Allenius BL, Cederfjäll C (2005). Quality of life, self-care ability, and sense of coherence in hemodialysis patients: a comparative study. Hemodialysis Int Symp Home Hemodialysis.

[19] Horsburgh ME, Rice VH, Matuk L (1998). Sense of coherence and life satisfaction: patient and spousal adaptation to home dialysis. ANNA J.

[20] Song YY, Chen L, Yu WW, Wang WX, Yang DJ, Jiang XL (2021). Correlates of symptom burden of hemodialysis patients. West J Nurs Res.

[21] Nahlén C, Saboonchi F (2010). Coping, sense of coherence and the dimensions of affect in patients with chronic heart failure. Eur J Cardiovasc Nurs.

[22] Lysandropoulos AP, Havrdova E (2015). 'Hidden' factors influencing quality of life in patients with multiple sclerosis. Eur J Neurol.

[23] Bragazzi NL. The gap in the current research on the link between health locus of control and multiple sclerosis: Lessons and insights from a systematic review. Multiple Sclerosis International. 2013;972471. 10.1155/2013/972471 PMC3586487 23476777

[24] McCabe MP, Stokes M, McDonald E (2009). Changes in quality of life and coping among people with multiple sclerosis over a 2 year period. Psychol Health Med.

[25] Klang B, Björvell H, Clyne N (1996). Quality of life in predialytic uremic patients. Qual Life Res.

[26] Uzdil N, Ceyhan Ö, Şimşek N (2022). The effect of salutogenesis-based care on the sense of coherence in peritoneal dialysis patients. J Clin Nurs.

[27] Kristofferzon ML, Engström M, Nilsson A (2018). Coping mediates the relationship between sense of coherence and mental quality of life in patients with chronic illness: A cross-sectional study. Qual Life Res.

[28] Kenne Sarenmalm E, Browall M, Persson LO, Fall-Dickson J, Gaston-Johansson F (2013). Relationship of sense of coherence to stressful events, coping strategies, health status, and quality of life in women with breast cancer. Psychooncology.

[29] Cohen J (1988). Statistical power analysis for the behavioral sciences.

[30] Lok P (1996). Stressors, coping mechanisms and quality of life among dialysis patients in Australia. J Adv Nurs.

[31] La VC (2018). place du psychologue en service de néphrologie-hémodialyse. Le Journal des Psychologues.

[32] Lindblad C, Sandelin K, Petersson LM, Rohani C, Langius-Eklöf A (2016). Stability of the 13-item sense of coherence (SOC) scale: a longitudinal prospective study in women treated for breast cancer. Qual Life Res.

[33] Eriksson M, Lindström B, Lilja J (2007). A sense of coherence and health. Salutogenesis in a societal context: Aland, a special case?. J Epidemiol Comm Health..

[34] World Health Organization. Process of translation and adaptation of instruments.: World Health Organization; 2020 [16–10–2020]. Available from: https://www.who.int/substance_abuse/research_tools/translation/en/

[35] Hays RD, Kallich J, Mapes D, Coons S, Amin N, Carter W, et al. Kidney Disease Quality of Life Short Form (KDQOL-SF), Version 1.3: A manual for use and scoring. Santa Monica, CA: Rand;1997.

[36] Peipert JD, Bentler PM, Klicko K, Hays RD (2018). Psychometric properties of the Kidney Disease Quality of Life 36-Item Short-Form Survey (KDQOL-36) in the United States. Am J Kidney Disease..

[37] Abd ElHafeez S, Sallam SA, Gad ZM, Zoccali C, Torino C, Tripepi G (2012). Cultural adaptation and validation of the “Kidney Disease and Quality of Life-Short Form (KDQOL-SF™) version 1.3” questionnaire in Egypt. BMC Nephrology..

[38] George D. SPSS for windows step by step: A simple study guide and reference, 17.0 update, 10/e: Pearson Education India; 2011.

[39] Schober P, Boer C, Schwarte LA (2018). Correlation coefficients: appropriate use and interpretation. Anesth Analg.

[40] Bendel RB, Afifi AA (1977). Comparison of stopping rules in forward “stepwise” regression. J Am Stat Assoc.

[41] Bursac Z, Gauss CH, Williams DK, Hosmer DW (2008). Purposeful selection of variables in logistic regression. Source Code Biol Med.

[42] Hosmer DWJ, Lemeshow S, Sturdivant RX. Model-building strategies and methods for logistic regression. Appl Logist Regress. 2013; 89–151.

[43] Mickey RM, Greenland S (1989). The impact of confounder selection criteria on effect estimation. Am J Epidemiol.

[44] Zhang Z (2016). Model building strategy for logistic regression: Purposeful selection. Ann Transl Med.

[45] Greimel E, Kato Y, Müller-Gartner M, Salchinger B, Roth R, Freidl W (2016). Internal and external resources as determinants of health and quality of life. PLoS ONE.

[46] Schult M-L, Ingrid S, Jacobs K (2000). The sense-of-coherence and the capability of performing daily occupations in persons with chronic pain. Work.

[47] Schnyder U, Büchi S, Mörgeli H, Sensky T, Klaghofer R (1999). Sense of coherence - A mediator between disability and handicap?. Psychother Psychosom.

[48] Flensborg-Madsen T, Ventegodt S, Merrick J (2006). Sense of coherence and physical health. The emotional sense of coherence (SOC-E) was found to be the best-known predictor of physical health. Sci World J.

[49] Cohen S (2004). Social relationships and health. Am Psychol.

[50] Sairenchi T, Haruyama Y, Ishikawa Y, Wada K, Kimura K, Muto T (2011). Sense of coherence as a predictor of onset of depression among Japanese workers: a cohort study. BMC Public Health.

[51] Siglen E, Bjorvatn C, Engebretsen LF, Berglund G, Natvig GK (2007). The influence of cancer-related distress and sense of coherence on anxiety and depression in patients with hereditary cancer: a study of patients' sense of coherence 6 months after genetic counseling. J Genet Couns.

[52] Veronese G, Pepe A (2014). Sense of coherence mediates the effect of trauma on the social and emotional functioning of Palestinian health providers. Am J Orthopsychiatry.

[53] Seligman ME, Csikszentmihalyi M (2014). Positive psychology: an introduction.

[54] Rangmar J, Sandberg AD, Aronson M, Fahlke C (2015). Cognitive and executive functions, social cognition and sense of coherence in adults with fetal alcohol syndrome. Nord J Psychiatry.

[55] Drageset J, Eide GE, Nygaard HA, Bondevik M, Nortvedt MW, Natvig GK (2009). The impact of social support and sense of coherence on health-related quality of life among nursing home residents–A questionnaire survey in Bergen, Norway. Int J Nurs Stud.

[56] Sadala ML, Miranda MG, Lorençon M, de Campos Pereira EP (2010). Nurse-patient communication while performing home dialysis: The patients' perceptions. J Ren Care.

[57] Paumier-Bidault L, Michel G (2018). Don de soins et dette de vie en hémodialyse. Topique.

[58] Riazuelo H, Cupa D, Chaudoye G (2014). Quand la dépendance est une question de survie: pratique clinique auprès de patients dialysés. Cliniques.

[59] Langius A, Lind MG (1995). Well-being and coping in oral and pharyngeal cancer patients. Eur J Cancer B Oral Oncol.

[60] Veenstra M, Moum T, Røysamb E (2005). Relationships between health domains and sense of coherence: a two-year cross-lagged study in patients with chronic illness. Qual Life Res.

[61] Bergman E, Malm D, Karlsson JE, Berterö C (2009). Longitudinal study of patients after myocardial infarction: sense of coherence, quality of life, and symptoms. Heart Lung: J Crit Care.

[62] Pusswald G, Fleck M, Lehrner J, Haubenberger D, Weber G, Auff E (2012). The, "Sense of Coherence" and the coping capacity of patients with Parkinson disease. Int Psychogeriatr.

[63] Eshel Y, Majdoob H, Goroshi M (2014). Posttraumatic recovery to distress symptoms ratio: a mediator of the links between gender, exposure to fire, economic condition, and three indices of resilience to fire disaster. Community Ment Health J.

[64] Delgado C (2007). Sense of coherence, spirituality, stress and quality of life in chronic illness. J Nurs Scholarsh.

[65] Surtees P, Wainwright N, Luben R, Khaw K-T, Day N (2003). Sense of coherence and mortality in men and women in the EPIC-Norfolk United Kingdom Prospective Cohort Study. Am J Epidemiol.

[66] Langeland N (2007). Time for specific diagnostics?. Tidsskrift for den Norske laegeforening: tidsskrift for praktisk medicin, ny raekke.

[67] Jean-Dit-Pannel R (2016). Narcisse à l’épreuve de l’hémodialyse: quelle méta-morphose?. Cliniques Méditerranéennes.

[68] Niihata K, Fukuma S, Akizawa T, Fukuhara S (2017). Association of coping strategies with mortality and health-related quality of life in hemodialysis patients: The Japan Dialysis Outcomes and Practice Patterns Study. PLoS ONE.

[69] Wileman V, Chilcot J, Armitage CJ, Farrington K, Wellsted DM, Norton S (2016). Evidence of improved fluid management in patients receiving haemodialysis following a self-affirmation theory-based intervention: A randomised controlled trial. Psychol Health.

[70] Crown S, Vogel JA, Hurlock-Chorostecki C (2017). Enhancing self-care management of interdialytic fluid weight gain in patients on hemodialysis: A Pilot study using motivational interviewing. Nephrol Nurs J.

[71] Pelikan JM, Dietscher C (2015). Why should and how can hospitals improve their organizational health literacy?. Bundesgesundheitsblatt Gesundheitsforschung Gesundheitsschutz.

[72] Golembiewski JA. Salutogenic architecture in healthcare settings. In: The handbook of salutogenesis. Springer: 2017:267–276. 28590652

[73] Zeinoun P, Akl EA, Maalouf FT, Meho LI (2020). The Arab region's contribution to global mental health research (2009–2018): a bibliometric analysis. Front Psych.

[74] Maalouf FT, Alamiri B, Atweh S, Becker AE, Cheour M, Darwish H (2019). Mental health research in the Arab region: challenges and call for action. Lancet Psychiatry.

[75] Kurita N, Wakita T, Ishibashi Y, Fujimoto S, Yazawa M, Suzuki T (2020). Association between health-related hope and adherence to prescribed treatment in CKD patients: multicenter cross-sectional study. BMC Nephrol.

[76] Kurita N, Wakita T, Fujimoto S, Yanagi M, Koitabashi K, Suzuki T (2021). Hopelessness and depression predict sarcopenia in advanced CKD and dialysis: a multicenter cohort study. J Nutr Health Aging.

[77] Lebanese Kidney Registry. Annual Report – 2012 Beirut: Lebanese Kidney Registry; 2012 [09 February 2022]. Available from: http://kidneyregistrylb.com/national-registry-2/annualreport-2012/.

[78] Ferrand A, Gorgos A, Ali N, Payot A (2018). Resilience rather than medical factors: How Parents predict quality of life of their sick newborn. J Pediatrics..

[79] Pasek M, Dębska G, Wojtyna E (2017). Perceived social support and the sense of coherence in patient–caregiver dyad versus acceptance of illness in cancer patients. J Clin Nurs.

[80] Antonovsky H, Sagy S (1986). The development of a sense of coherence and its impact on responses to stress situations. J Soc Psychol.

[81] Bowman BJ (1996). Cross-cultural validation of Antonovsky's Sense Of Coherence Scale. J Clin Psychol.

[82] Nosheen A, Riaz M, Batool N (2014). Cross-cultural study on social support, sense of coherence and outcomes in Pakistan and Germany. Pak J Commerce Soc Sci (PJCSS).

